# Bacterial community and genome analysis of cytoplasmic incompatibility-inducing *Wolbachia* in American serpentine leafminer, *Liriomyza trifolii*

**DOI:** 10.3389/fmicb.2024.1304401

**Published:** 2024-02-06

**Authors:** Ajeng K. Pramono, Ardhiani K. Hidayanti, Yohsuke Tagami, Hiroki Ando

**Affiliations:** ^1^Laboratory of Phage Biologics, Graduate School of Medicine, Gifu University, Gifu, Japan; ^2^School of Biological Environment, The United Graduate School of Agricultural Science, Gifu University, Gifu, Japan; ^3^School of Life Sciences and Technology, Institut Teknologi Bandung (ITB), Bandung, Indonesia; ^4^Laboratory of Applied Entomology, Faculty of Agriculture, Shizuoka University, Shizuoka, Japan; ^5^Center for One Medicine Innovative Translational Research (COMIT), Gifu University, Gifu, Japan

**Keywords:** *Wolbachia*, *Liriomyza trifolii*, CIF, cytoplasmic incompatibility factor, phage WO

## Abstract

*Liriomyza trifolii*, an agricultural pest, is occasionally infected by *Wolbachia.* A *Wolbachia* strain present in *Liriomyza trifolii* is associated with cytoplasmic incompatibility (CI) effects, leading to the death of embryos resulting from incompatible crosses between antibiotic-treated or naturally *Wolbachia*-free strain females and *Wolbachia*-infected males. In this study, high-throughput sequencing of hypervariable rRNA genes was employed to characterize the bacterial community in *Wolbachia*-infected *L. trifolii* without antibiotic treatment. The analysis revealed that *Wolbachia* dominates the bacterial community in *L. trifolii*, with minor presence of *Acinetobacter*, *Pseudomonas*, and *Limnobacter*. To elucidate the genetic basis of the CI phenotype, metagenomic sequencing was also conducted to assemble the genome of the *Wolbachia* strain. The draft-genome of the *Wolbachia* strain *w*Ltri was 1.35 Mbp with 34% GC content and contained 1,487 predicted genes. Notably, within the *w*Ltri genome, there are three distinct types of cytoplasmic incompatibility factor (*cif*) genes: Type I, Type III, and Type V *cifA;B*. These genes are likely responsible for inducing the strong cytoplasmic incompatibility observed in *L. trifolii*.

## 1 Introduction

*Wolbachia* are intracellular symbiont bacteria (Phylum: Pseudomonadota, Class: Alphaproteobacteria) found in various terrestrial arthropods and nematodes. About 20–66% of both animal taxa are infected by *Wolbachia* (Hilgenboecker et al., 2008; Gomes et al., 2022). The maternally inherited endosymbiotic *Wolbachia* can manipulate the reproduction of their hosts, using several mechanisms, including cytoplasmic incompatibility (CI), male killing, parthenogenesis, and feminization (Correa and Ballard, 2016). CI causes offspring death when an infected male mates with an uninfected female, while the mating of infected females with the same *Wolbachia* strain produces viable offspring either way (Yen and Barr, 1973; Shropshire et al., 2020). The degree of CI induction can vary significantly between different *Wolbachia* strains, with some strains causing no reproductive manipulation or CI, such as *w*Au in *Drosophila simulans* and *w*Mau in *D. mauritiana*, while others can cause weak CI as in *w*Yak of *D. yakuba*, or complete CI that affects all embryos in *w*Pip of *Culex pipiens* complex species (Laven, 1967; Hoffmann et al., 1996; Meany et al., 2019; Beckmann et al., 2021). CI induced by *Wolbachia* can be a useful technique for controlling insect populations. The use of CI-*Wolbachia* has been effective in controlling the mosquito vector-borne diseases population, reducing the transmission of disease in the health sector, and can be considered in the agricultural sector through incompatible insect technique (IIT) to control insect pests (Laven, 1967; Hidayanti et al., 2022).

Cytoplasmic incompatibility is a two-sided phenomenon, involving a form of “modification” in sperm and a corresponding “rescue” mechanism occurring within the eggs (Hurst, 1991). The CI effect in *Wolbachia* is mainly attributed to a class of genes known as cytoplasmic incompatibility factor (*cif*) genes, which forms the molecular basis of CI (Beckmann et al., 2017, 2019; Le Page et al., 2017). The *cif* genes, *cifA* and *cifB*, usually occur in an operon, but unpaired and fragmented *cif* genes are also found in some strains (Martinez et al., 2021). In addition, multiple pair’s amplification and diversification of *cif* genes have been reported to contribute to CI diversity in *w*Pip from the mosquito *Culex pipiens* (Bonneau et al., 2018a,b). The *cif* gene products are categorized into Type I–V based on their protein domain similarity (Lindsey et al., 2018; Martinez et al., 2021). Cif proteins with deubiquitinase activity are sometimes referred to as Cid, while Cif proteins with DNase activity are sometimes referred to as Cin (Beckmann et al., 2017). The affinity between CidA–CidB, and CinA–CinB, have been confirmed (Beckmann et al., 2017; Chen et al., 2019). Furthermore, co-expressing *cifA*/*cifB* transgenes in *Drosophila melanogaster* also mimics the embryonic defects, a feature of CI that results in embryo death (Le Page et al., 2017; Chen et al., 2019), while transgenic expression of *cifB* alone induces CI in *Anopheles gambiae* (Adams et al., 2021). Besides, transgenic expression of a single *cifA* gene can rescue defects in egg-hatch rates and growth defects induced by CifB in yeast (Shropshire et al., 2018; Adams et al., 2021).

*Liriomyza trifolii* (Diptera: Agromyzidae), a polyphagous leafminer insect is a significant invasive pest of agricultural vegetable and ornamental plants (Kang et al., 2009; Zhang et al., 2017). *Wolbachia* infection is prevalent in *Liriomyza* species in Japan and the Indo-Pacific region, with 30–80% of the population being positive for the infection (Xu et al., 2021). *Wolbachia*-infected *L. trifolii* exhibited strong CI phenotype, resulting in very few eggs hatching from the crossing between infected males with naturally *Wolbachia*-free or antibiotic-treated females (Tagami et al., 2006a). The *Wolbachia* strain found in *L. trifolii* has been assigned to Supergroup B, in contrast to the majority of *Wolbachia* strains identified in Diptera, which belong to Supergroup A (Scholz et al., 2020). Unfortunately, the low completeness of the initial genome has hindered the identification of *cif* genes and other important gene markers, such as the *Wolbachia* surface protein gene *wsp* (Braig and Zhou, 1998) and the five housekeeping genes (*gatB*, *coxA*, *hcpA*, *ftsZ*, and *fbpA*) used in the *Wolbachia* multilocus strain typing (MLST) methodology (Baldo et al., 2006).

In this study, we surveyed the bacterial community in *L. trifolii* to explore its microbiome, and sequenced, assembled, and analyzed the genome of its *Wolbachia* to investigate the putative genetic basis of the strong CI effect on its insect host. We additionally provided a detailed description of the newly assembled genome through phylogenetic and comparative genome analysis. This involved the identification of *cif* genes and the prophage region to further characterize the strains in comparison to other closely related *Wolbachia* genomes.

## 2 Materials and methods

### 2.1 Insect rearing, sample collection, and DNA extraction

*Liriomyza trifolii* were provided by Applied Entomology Laboratory, Faculty of Agriculture, Shizuoka University. The flies were isolated in Hamamatsu, Shizuoka, Japan in 1991, and maintained on the leaves of kidney bean plants in a 34 cm (width) × 35 cm (length) × 34 cm (height) cage with light–dark regime (16:8) at 23°C (Tagami et al., 2006a; Hidayanti et al., 2022).

Total genomic DNA was extracted from *L. trifolii* using Qiagen DNeasy Blood and Tissue Kit, following the manufacturer’s instruction (Qiagen, Hilden, Germany), with slight modifications. Adults insects (*n* = 30–50) were crushed in 50 μL ATL buffer with a motorized pestle (Power Masher II; Nippi, Tokyo, Japan); 130 μL of ATL buffer, and 20 μL proteinase-K (20 mg/mL) were added to the homogenate and incubated in a 56°C dry bath incubator (Major Science, Taiwan) for 2–3 h; 200 μL of buffer AL and 200 μL of 99.5% ethanol were added following incubation at 56°C; to maximize DNA yield, the DNA was eluted in 50 μL of buffer AE. DNA yield and purity were verified with a Nanodrop 1000 spectrophotometer (Thermo Fisher Scientific, Waltham, MA, United States). The extracted DNA was aliquoted for sequencing and cloning of 16S rRNA gene and the *Wolbachia* surface protein (*wsp*) gene, and metagenomic library construction for next-generation sequencing (NGS).

### 2.2 High-throughput and sanger amplicon sequencing

#### 2.2.1 High-throughput 16S rRNA amplicon sequencing

The total DNA from *L. trifolii* was amplified using primers to target the hypervariable V3–V4 regions of the 16S rRNA gene, which includes Illumina adaptor sequences (in triplicate), using these primers: Forward Primer (5′-TCGTCGGCAGCGTCAGATGTGTATAAGAGACAGCCTACGGGNGGCWGCAG-3′) and Reverse Primer (5′-GTCTCGTGGGCTCGGAGATGTGTATAAGAGACAGGACTACHVGGGTATCTAATCC-3′). The amplicon classification was also confirmed with the near-full-length 16S rRNA gene clones sequencing, amplified using primers 27F-mix (5’-AGRGTTTGATYMTGGCTCAG-3′) and 1492R (5’-GGHTACCTTGTTACGACTT-3′) (Hongoh et al., 2007).

The high-throughput amplicon sequencing analysis was performed using the QIIME2-2021.4 platform (Bolyen et al., 2019). Adapter and primer sequences were removed using the following options: --p-front-f CCTACGGGNGGCWGCAG --p-front-r GACTACHVGGGTATCTAATCC. The reads were trimmed, denoised, paired, and dereplicated with the built-in dada2 algorithm (Callahan et al., 2016). The amplicon sequence variants (ASVs) were taxonomically classified using the q2-feature-classifier, which had been trained for V3–V4 regions of 16S rRNA (Bokulich et al., 2018).

#### 2.2.2 *Wolbachia* amplicon sequencing

The presence of *Wolbachia* was detected using *Wolbachia* surface protein (*wsp*) gene primers (wsp81F: 5’-TGGTCCAATAAGTGATGAAGAAAC-3′ and wsp691R: 5’-AAAAATTAAACGCTACTCCA-3′) (Braig and Zhou, 1998). The *wsp* amplicons were cloned and sequenced to confirm whether the *Wolbachia* strain in the current sample was identical to the previously detected *Wolbachia* associated with strong cytoplasmic incompatibility in *L. trifolii* (Tagami et al., 2006a). The alignment of the *wsp* sequences was also performed with the previously sequenced *wsp* from the *Wolbachia* survey in Japan and Indo-pacific region (Xu et al., 2021).

### 2.3 *Wolbachia* shotgun sequencing, genome assembly and bioinformatics analyses

The *Wolbachia* draft genome was obtained by preparing a shotgun metagenome library with an Illumina DNA prep kit, which was then sequenced using the MiSeq Reagent Kit v2 and v3. The metagenomic sequencing reads were filtered based on base quality (Q20) and length (>50 bp). Next, the filtered reads were paired and assembled using MEGAHIT (Li et al., 2015) and only contigs longer than 1,000 bp were retained for further analysis. A custom database of *Wolbachia* genomes was utilized to identify *Wolbachia* contigs based on nucleotide similarity using blastn (Altschul et al., 1990). Scaffolds were created using Codon Code Aligner (v. 9.0.1, Codon Code Corporation) and Mauve Contig Mover (MCM) (Rissman et al., 2009), followed by manual inspection and visualization in Geneious Prime 2022.1.1. Genome completeness was determined by the presence of single-copy genes of Proteobacteria (proteobacteria_odb10) using BUSCO 5.2.2 (Manni et al., 2021). Average Nucleotide Identity (ANI) was calculated using FastANI tool (Jain et al., 2018). Annotation of coding regions, RNA genes, and other genomic features was done through the RAST-tk pipeline (Aziz et al., 2008). The prophage region was automatically detected using PHASTER (Arndt et al., 2016) and subsequently refined manually based on similarity searches against prophage region in the *Wolbachia* of *Ischnura elegans*, the largest *Wolbachia* genome assembled from the Darwin Tree of Life biodiversity genomics project (Vancaester and Blaxter, 2023). Identification of Gene Transfer Agents (GTAs) was also performed through sequence similarity searched against RcGTA in *Rhodobacter capsulatus* and a putative GTA in *w*Mel. The sequence similarity searches were conducted using the BLAST suite against the nr database (https://www.ncbi.nlm.nih.gov/), a custom *Wolbachia* genome database (Supplementary Table S1; Pascar and Chandler, 2018), or specific genes as otherwise mentioned.

### 2.4 Phylogenomic analysis

A phylogenetic tree of *Wolbachia* was created from concatenated 40 single copy orthologous genes, aligned with MAFFT 7.402 (Katoh and Standley, 2013). The trees were generated with 1,000 bootstraps using Maximum Likelihood method and JTT matrix-based model in MEGAX (Kumar et al., 2018). For the CI genes phylogeny, the *cifA* and *cifB* homologs were identified and constructed as previously described (Martinez et al., 2021). To clarify the origin of the large terminase (*terL*) in *w*Meg, an unrooted *terL* phylogenetic tree was constructed using identified homologs from reference genomes. These homologs were retrieved through nucleotide similarity searches, using the following sequences as queries: the *terL* sequences downstream of the Type III *cifA;B* in *w*Meg (CP021120.1), and the previously identified *terL* sequences in WORiC (CP001391.1; sr1WO), WORiA (CP001391.1; sr2WO), WOMelB (AE017196.1; sr3WO), as well as WOFol2 (CP015510.2; sr4WO).

### 2.5 Protein domain prediction

The *cif* genes were translated into amino acid then queried individually using HHpred (https://toolkit.tuebingen.mpg.de/hhpred/; Söding et al., 2005) with defaults parameters against the following databases: PDB_mmCIF70_24_Oct, SCOPe70_2.08, Pfam-A_v36, SMART_v6.0, PHROGs_v4, TIGRFAMs_v15.0, and COG_KOG_v1.0.

## 3 Results

### 3.1 Microbiome of *Liriomyza trifolii*

The *Wolbachia* strains in the current study was identical to the strain associated with strong CI in *L. trifolii* in the previous study (Tagami et al., 2006a). The identity was confirmed using a partial *wsp* gene which was amplified, cloned, and Sanger-sequenced (*n* = 24). The *wsp* gene is commonly used as an indicator of *Wolbachia* infection in insects. There are at least three *wsp* alleles have been identified in *Wolbachia* in *L. trifolii*, *w*LtriA, *w*LsatA, and *w*LsatD. All clones from the sample were identical to the *w*LsatD-type allele, hereafter referred to as *w*Ltri.

High-throughput sequencing of the hypervariable region of the 16S rRNA gene was also performed to gain insights into the bacterial community present in *L. trifolii* without the antibiotic treatment. A total of 533,982 V3–V4 amplicon reads were quality-filtered, de-noised, and merged into 176,493 functional sequences. These sequences were de-replicated into 161 ASVs, in which 28 ASVs accounted for more than 80% of the total reads. Each of these ASVs were classified into taxa; in contrast to operational taxonomic unit (OTU)-based clustering, different ASVs could be classified into the same taxon.

**Figure 1:**
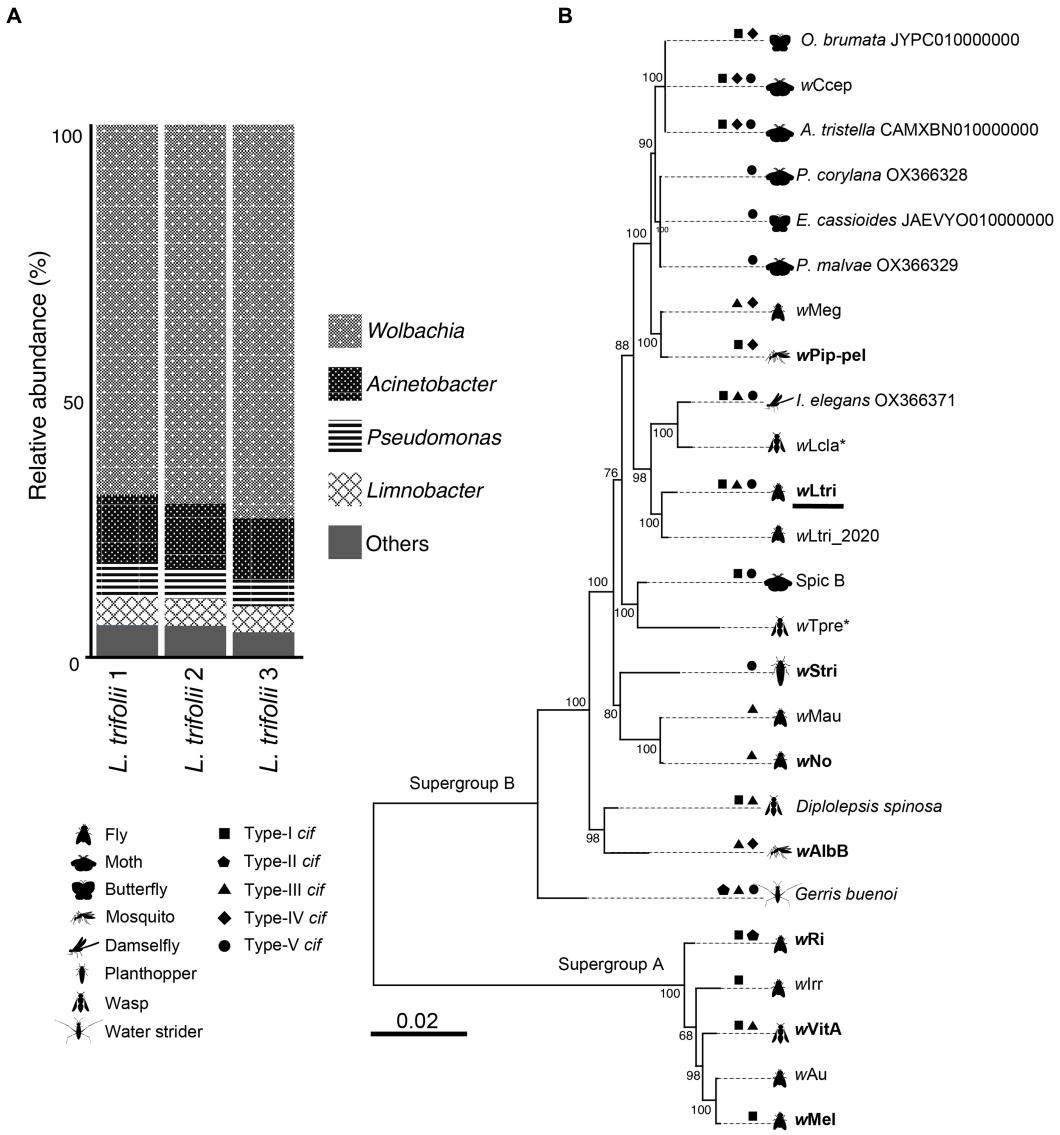
**(A)** Prokaryote composition in *Liriomyza trifolii* at the genus level in triplicate. **(B)** Phylogeny of 25 strains of *Wolbachia*. The tree was constructed based on nucleotide sequences of 40 single-copy orthologous genes. The *Wolbachia* strains that are reported to cause CI are presented in boldface, while the parthenogenesis-inducing strains are marked with an asterisk. The *w*Ltri from this current study is underlined.

The bacterial community in *L. trifolii* was largely dominated by Pseudomonadota (synonym, Proteobacteria) members, accounting for 96% of the total ASVs, with a minor presence of Actinobacteria, Bacteroidota, and Firmicutes. Among the identified genera, *Wolbachia* was the most abundant taxon (Figure 1A), with the representative ASV exhibiting an identical sequence to that of CI-inducing *Wolbachia* strains which infect a rice moth, *Corcyra cephalonica* (wCcep), as well as *Wolbachia* strains derived from other moths, such as *Agriphila tristella*, *Erebia cassioides*, and *Operophtera brumata*. The cloning and sequencing of an almost full-length 16S gene confirmed this finding. Likewise, ASVs from *Acinetobacter* and *Pseudomonas* were detected and matched to the 16S rRNA sequences recovered from metagenomic assembled contigs. These genera have been consistently reported to co-exist with *Wolbachia* in both wild and laboratory-reared mosquitoes, including *Culex* and *Aedes* (Beier et al., 1996; Pidiyar et al., 2002, 2004; Lindh et al., 2005; Zouache et al., 2009a,b; Minard et al., 2013; Schrieke et al., 2021; Rau et al., 2022).

### 3.2 The genome of *w*Ltri, the *Wolbachia* strain in *Liriomyza trifolii*

**Figure 2:**
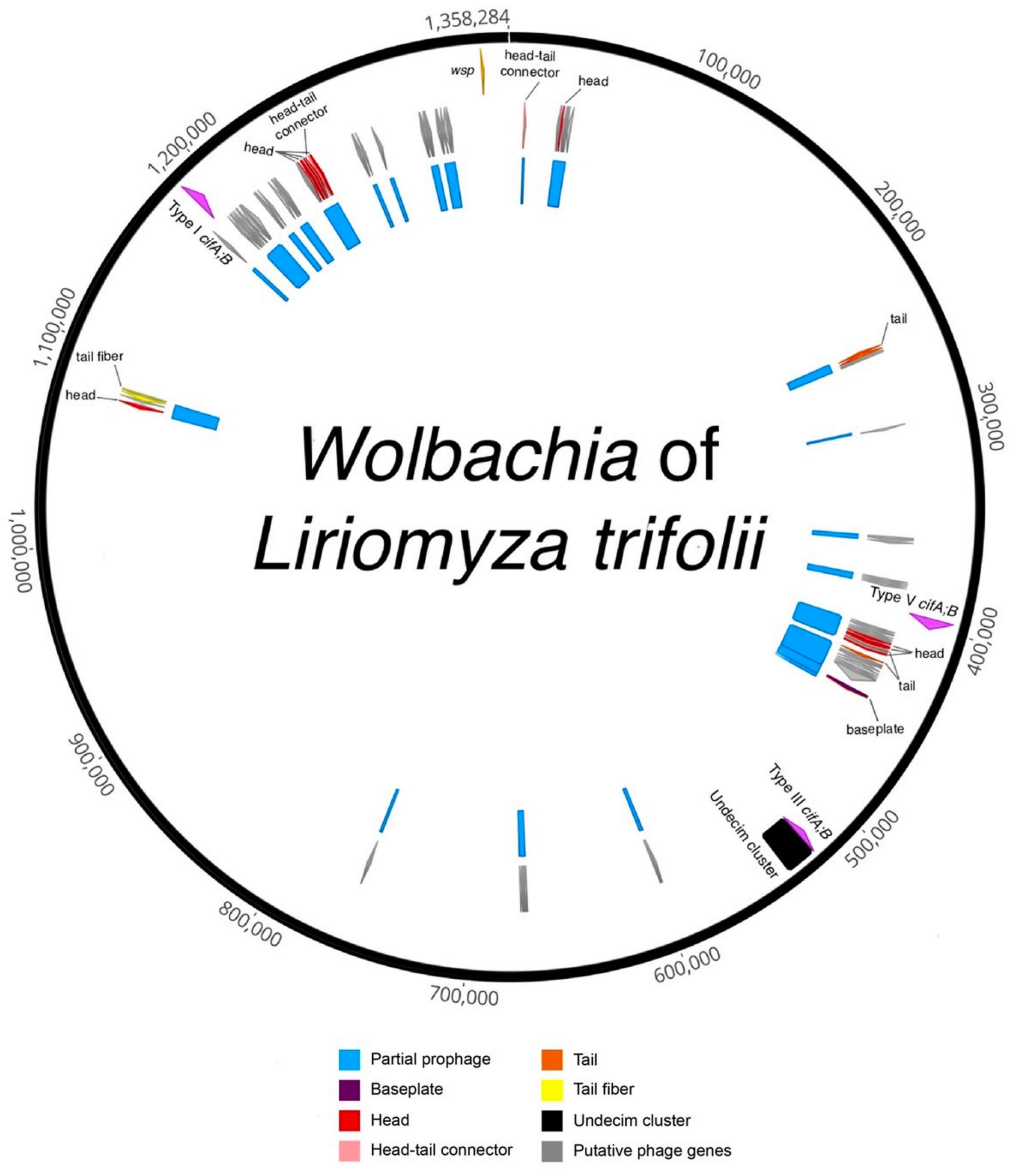
Circularized draft genome annotation of the *Wolbachia* of *Liriomyza trifolii.* Partial prophage regions are indicated in blue, representing regions with incomplete prophage regions predicted by PHASTER and/or parts of prophage WO in the *Wolbachia* of *Ischnura elegans*. Structural genes are denoted in red (head), pink (head-tail connector), purple (baseplate), orange (tail), and yellow (tail fiber), while gray signifies putative phage genes, encompassing genes found in phages but with unknown functions. The Undecim cluster is depicted in black. The *cif* genes are highlighted in fuchsia, and the *Wolbachia* surface protein (*wsp*) gene is represented in light orange.

A total of 15,421,862,646 high-quality bases were assembled using MEGAHIT into contigs. A contig covering the entire *L. trifolii* mitochondrial sequence was identified, and it exhibited 99.1% nucleotide similarity to *L. trifolii* GU327644. A total of 435 contigs originating from *Wolbachia* were identified using BLASTN searches against a custom database (Supplementary Table S1) and further validated via manual inspection. The *w*Ltri contigs were reordered using Mauve Contig Mover (MCM) to create a draft genome (Figure 2). The genome comparison analysis also included a previously sequenced *L. trifolii*, which contains 443 contigs of *Wolbachia*, assembled using a similar short-read sequencing technology and genome assembler, referred to as *w*Ltri_2020 hereinafter (Vicoso and Bachtrog, 2015; Scholz et al., 2020). The Average Nucleotide Identity score between *w*Ltri draft genome and wLtri_NCBI was 98.1%. The completeness of the *w*Ltri genome based on BUSCO (proteobacteria_odb10) was 80.3%, which represents a typical value for complete *Wolbachia* genomes (Sinha et al., 2019), while the completeness of *w*Ltri_2020 was only 31.6%. The assembly size of *w*Ltri was 1,358,284 bp, which was longer than that of *w*Ltri_2020 (879,722 bp). The genome of *w*Ltri contained 1,487 coding sequences (CDSs), along with 5S, 23S, and 16S rRNA, and 34 tRNA genes, while *w*Ltri_2020 had fewer CDSs (*n* = 1,206) and an incomplete set of rRNA genes (Table 1).

**Table 1:** Comparison of assembly status and genome characteristics among *Wolbachia* strains.

*Wolbachia* strain	Insect host	Total length (bp)	Contigs	GC%	CDSs	tRNAs	rRNAs	BUSCO score^*^	Reference
*w*Ltri	*Liriomyza trifolii*	1,358,284	435	34.0	1,487	34	3	80.3	This study
*w*Ltri_2020	*Liriomyza trifolii*	817,747	443	33.5	1,206	18	1	31.6	Scholz et al. (2020)
*w*Meg	*Chrysomya megacephala*	1,376,868	1	34.0	1,242	34	3	85.5	Unpublished (2019)
*w*Pip-pel	*Culex quinquefasciatus*	1,482,455	1	34.2	1,373	34	3	86.3	Klasson et al. (2008)
*w*VitA	*Nasonia vitripennis*	1,325,529	142	32.3	1,325	34	3	83.9	Newton et al. (2016)
*w*Di	*Diaphorina citri* Kuwayama	1,528,786	1	34.1	1,394	34	3	80.6	Neupane et al. (2022)

^*^Based on proteobacteria_odb10. CDS, Coding sequence.

Based on the maximum likelihood phylogenetic tree of single-copy genes, the newly sequenced *w*Ltri belongs to the Supergroup B and it clustered with *w*Ltri_2020, which infects the same host species, *L. trifolii* (Figure 1B)*. Wolbachia* of Supergroup B is commonly found in Lepidopteran hosts, with only a few instances identified in Dipteran hosts (Meany et al., 2019; Scholz et al., 2020; Vancaester and Blaxter, 2023). Within the Supergroup B clade, *w*Ltri formed a monophyletic group with *Wolbachia* strains that infect other arthropods, such as damselflies (*I. elegans*) and wasps (*Leptopilina clavipes*), rather than with strains infecting flies, such as *w*Mau of *Drosophila mauritiana* and *w*No of *Drosophila simulans* (Figure 1B). To further investigate the relationship between *w*Ltri and *Wolbachia* strains in wasps, a phylogenetic tree was constructed using *wsp* genes (Supplementary Figure S1). The analysis included a parasitoid wasp, *Hemiptarsenus* var*icornis*, found near the initial *L. trifolii* sampling location (Tagami et al., 2006b). Although the *wsp* gene undergoes rapid evolution, the *wsp* gene derived from *w*Ltri consistently clustered with the *wsp* amplified from wasps *H. varicornis* and *Trichogramma pretiosum* (wTpre).

### 3.3 Prophage regions in *w*Ltri

In the case of *w*Ltri, prophage sequences were analyzed using the PHASTER tool and sequence homology to previously known prophage WO to identify incomplete prophage regions with a combined size of 98.9 Kb (Figure 2). Prophage regions were also identified using nucleotide similarity searches against *Wovirus*, which includes WO phages, from a newly proposed family, Symbioviridae. The *Wovirus* was further subclassified into four groups, sr1WO, sr2WO, sr3WO, and sr4WO, based on gene synteny in the phage core module and serine-recombinase nucleotide identity (Bordenstein and Bordenstein, 2022). A BLASTN search using these recombinases revealed fragments in wLtri with 84 and 88% nucleotide similarity to sr1WO and sr3WO, respectively.

Our analysis also revealed the presence of a set of 11 conserved genes, known as Undecim Cluster, in *w*Ltri genome. This cluster is part of the EAM of Phage WO, which is commonly found in sr3WO and is occasionally present in sr4WO and WO-like islands (Bordenstein and Bordenstein, 2022). The EAM in *w*Ltri displays a module synteny similar to that of the WO-like island in WOAlbB3, *w*No, WOMau2, and *w*VitA, where it also comprises *cifA* and *cifB* genes (Bordenstein and Bordenstein, 2022). Notably, the *cif* genes found in these WO-like islands belong to Type III *cifA;B*, unlike in sr3WO, which mostly includes Type I *cifA;B*.

Interestingly, in *w*Meg, downstream of Undecim cluster—Type III *cifA;B*, a large terminase gene (*terL*) was identified (Supplementary Figure S2A). The gene is commonly used as a prophage marker due to its high degree of conservation and ubiquity across phage genomes. To determine whether the *terL* was originated from a Phage WO carrying the Type III *cifA;B*, the intergenic region between the *cif* genes and *terL* was aligned with other *Wolbachia* which possessed same type of *cif* genes in the reference genomes, including *w*Ltri. The intergenic region (1,113 bp) displayed potential shared synteny (Supplementary Figure S2B), revealing distinct partitioning into two segments: a left portion (L; 554 bp) downstream of the Undecim cluster—*cif* genes, and a right portion (R; 559 bp) upstream of *terL*. While both segments were occasionally repeated in some of the reference genomes, they were never co-occurred, except in *w*Meg (Supplementary Figure S2C; Supplementary Tables S2, S3).

The L was found adjacent to *cif* genes and/or the Undecim Cluster in the genomes that possess it. In the *Wolbachia* of *I. elegans*, the L was identified in two locations: the first near a partial Undecim Cluster—*cif* genes, and the second near an incomplete Undecim Cluster without *cif* genes in a different location (Supplementary Table S2). In *w*Irr, the L was associated with IS256, while in the *Wolbachia* of *Erebia cassioides* and *Leptopilina clavipes*, *w*Lcla, it was associated with IS110 family transposase (Supplementary Table S2). Conversely, the R was found in more location within the genomes, often at breakpoints, and was associated with IS982, IS5, and IS630 (Supplementary Figure S3; Supplementary Table S3).

In the *Wolbachia* genome, a terminase gene may also have been derived from Gene Transfer Agents (GTAs). BLAST searches (TBLASTN, BLASTN) using the amino acid and nucleotide sequences of a terminase-like protein (RCAP_rcc01683) from RcGTA, a well-studied GTA in *Rhodobacter capsulatus*, as queries against the *Wolbachia* reference genomes and *w*Ltri, did not yield any significant matches. However, when a terminase from the putative GTA in *w*Mel (WD_1016; AE017196) was used as a query, it revealed matches within the reference genomes (Supplementary Table S4). Notably, these findings included the *terL* which was located downstream of the WO-like island in *w*Meg, revealing 82% nucleotide and 83% amino acid similarities, respectively. In addition, a phylogenetic tree constructed using homologs of *terL* from *w*Meg and prophage WO revealed that they belong to two distinct clades, which further divided into a sub-clade that separates Supergroup A from Supergroup B (Supplementary Figure S2C).

### 3.4 Cytoplasmic incompatibility genes

**Figure 3:**
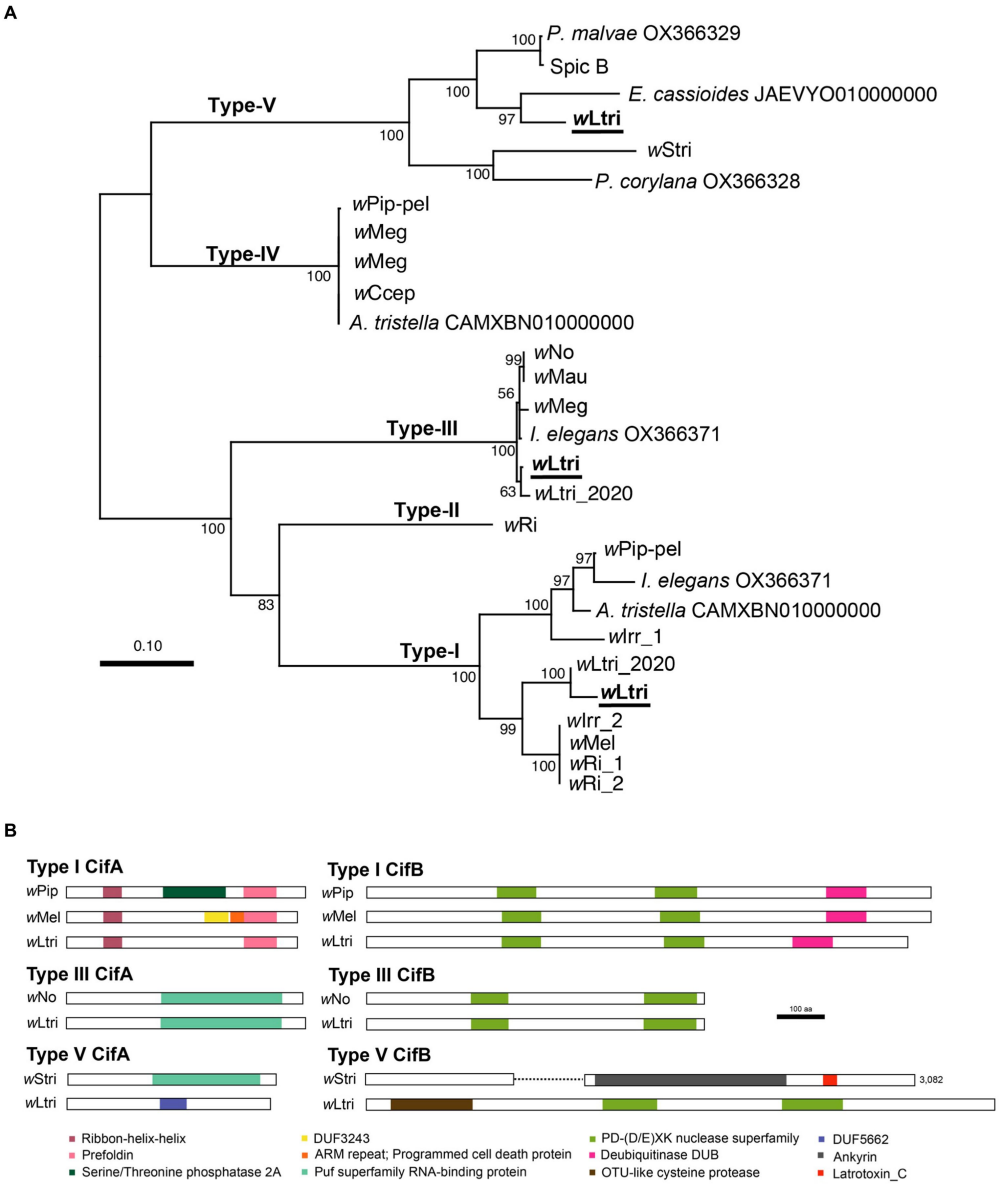
**(A)** Maximum likelihood tree of concatenated *cifA* and *cifB* nucleotide sequences. Partially sequenced *cif* homologs were excluded. Bootstrap values were estimated from 1,000 replicates. **(B)** Representative structures of Cif proteins with predicted domains. The *w*Stri type V CifB has a length of 3,082 amino acids. To accommodate presentation constraints, it was shortened without eliminating any identifiable domains.

*w*Ltri has three phylogenetically distinct *cifA–cifB* gene pairs which belong to Type I, III, and V (Figure 3A). The *w*Ltri Type I showed moderate to low amino acid similarity to the CifA (63–68%) and CifB (48–51%) of the experimentally validated CI-inducing *Wolbachia* strains *w*Mel and *w*Pip, respectively. The Type V also showed moderate and low amino acid similarity of 57 and 20% to the CifA–CifB proteins of *w*Stri, respectively. On the other hand, *w*Ltri Type III showed high amino acid similarity (98%) to CifA and CifB of CI-inducing *w*No. Furthermore, the *w*Ltri Type III was the only gene pair located within the EAM of WO-like islands, adjacent to an Undecim cluster.

In *w*Ltri CifA, four protein domains were identified (Figure 3B). The first two were the Ribon-helix–helix Protein (RHH domain) and Prefoldin, found in the Type I CifA of *w*Ltri. Despite a low probability of homology (probability 22–31%), these domains were also found in functional CifAs (CidA). However, the CifA of *w*Ltri lacks Serine/Threonine phosphatase 2A and DUF3243, which are present in *w*Pip and *w*Mel, respectively. Additionally, the Puf superfamily RNA-binding protein (Type III) was identified in both *w*Ltri and *w*No. Lastly, the DUF 5662 (Type V) was found in *w*Ltri but not in *w*Stri Type V CifA, which contains the Puf superfamily. In *w*Ltri CifB, three protein domains were identified (Figure 3B). The PD-(D/E)XK nuclease superfamily was consistently found in all *w*Ltri CifB with a high probability (>96%). Furthermore, the Type I exhibited the presence of a deubiquitinase domain DUB (probability 96%), while the Type V contained an OTU-like cysteine protease (probability 99%).

## 4 Discussion

This study represents the first survey of the bacterial community of the American Serpentine Leafminer fly, *L. trifolii*, in Japan. Using 16S rRNA high-throughput amplicon sequencing, it revealed *Wolbachia* as the most abundant bacterium in *L. trifolii*. The genome of the *Wolbachia* strain, *w*Ltri, had never been assembled with high completeness before. This *w*Ltri assembly represents the most complete genome sequence of a cytoplasmic-inducing *Wolbachia* of *L. trifolii*. Another available *Wolbachia* genome from *L. trifolii*, *w*Ltri_2020, is a binning of 443 contigs with only 31.6% BUSCO completeness (Vicoso and Bachtrog, 2015; Scholz et al., 2020). *w*Ltri_2020 was not included in some of the analyses in this study as it contains only partial genome information. We have also determined that *w*Ltri was the main endosymbiont of *L. trifolii*, using16S rRNA gene amplicon sequencing and metagenomic data.

Unlike the majority of *Wolbachia* strains found in Dipteran hosts, which belong to Supergroup A, *w*Ltri is classified under Supergroup B. The phylogeny of supergroup A and B have been found to be incongruent with those of their hosts, due to frequent horizontal transmission of *Wolbachia* strains across diverse host species (Raychoudhury et al., 2009; Wang et al., 2020). Insect parasitoids have been proposed as a means of facilitating this horizontal transmission of endosymbionts when infected and uninfected parasitoid wasps develop within the same host insect (Huigens et al., 2000, 2004; Ahmed et al., 2015). *Liriomyza trifolii* is also susceptible to parasitoid wasps, with approximately 24 species of leafminer parasitoids identified in Japan (Arakaki and Kinjo, 1998). Among these wasps, a *wsp* sequence from *H.* var*icornis*, found near the original sampling location of *L. trifolii*, formed a clade with that of *w*Ltri. However, due to the limited availability of *wsp* sequences from other parasitoids, it was the only sequence included in the analysis. This limitation is attributed to the lower prevalence of *Wolbachia* infection in leafminer parasitoids compared to *Liriomyza*, in which among the surveyed 15 leafminer parasitoid species, only *H.* var*icornis* was infected with *Wolbachia* (Tagami et al., 2006b). Besides, the migration of leafminers *Liriomyza* from another country (Abe, 2017) may potentially introduce endosymbiont transfer between the established *Liriomyza* and the invasive species. Therefore, additional research is needed to comprehensively understand the potential role of *Liriomyza* parasitoids and the impact of invasive *Liriomyza* on the horizontal transmission of *Wolbachia* strains.

*Wolbachia* is well known for inducing CI in many insects, including *L. trifolii* (Tagami et al., 2006a). Recent studies have shown that the proteins which are responsible for CI, CifA and CifB, can be classified into Types I–V (Martinez et al., 2021). In the genome of *w*Ltri, three sets of *cifA;B* genes—Types I, III, and V—were identified. Notably, the genes encoding Type I and Type V CifB in *w*Ltri were shorter, exhibiting low protein similarity to functional Cif in *w*Pip, *w*Mel, *w*No, and *w*Stri. Typically, *cifB* often accumulates more mutations before *cifA*, rendering the gene non-functional before being eliminated from the genome (Martinez et al., 2021). However, in *w*Ltri, although the genes were shorter, the predicted gene products containing domains commonly found in Type I CifB, such as PD-(D/E)XK nuclease superfamily and DUB, remained recognizable. The Type V CifB in *w*Ltri was also considerably shorter than that of *w*Stri, a *Wolbachia* strain in *Laodelphax striatellus*. This difference is not unexpected due to the greater diversity of protein domains in Type V CifB compared to other types, encompassing domains such as the C-terminal domain of Latrotoxin, those involved in protein–protein interactions (tetratricopeptide and ankyrin repeats), and a protease domain (OTU-like cysteine protease) (Martinez et al., 2021). However, in *w*Ltri, only the latter and a PD-(D/E)XK were present. In contrast, the Type III *cifA;B* of *w*Ltri appeared highly conserved, sharing adjacent gene synteny with CI-inducing *Wolbachia* strains like *w*No, which exclusively contains Type III *cifA;B* genes.

The amplification-diversification of functional *cif* genes and the cumulative presence of these genes have been associated with cytoplasmic incompatibility (CI) strength (Le Page et al., 2017; Bonneau et al., 2018b). In the genome of *Wolbachia* in *Culex pipiens w*Pip, variations and copy numbers of *cif* genes (*cidA*, *cidB*) are identified and the expression of these multiple *cid* gene variants in males may account for differences in CI cellular phenotypes (Bonneau et al., 2018a,b). Furthermore, *w*Mel, a *Wolbachia* strain with only one copy of these genes exhibits a weak CI phenotype, whereas strains with two or three copies of the genes, such as *w*Ri and *w*Ha, showed a strong CI effects (Le Page et al., 2017). The facts that *w*Ltri causes strong CI in *L. trifolii* (Tagami et al., 2006a) and its genome harbored three sets of *cifA–cifB* genes, might supported this correlation. Ultimately it is necessary to confirm that these *cif* gene products interact with each other through performing an *in vitro* pull-down study, which demonstrates the specific binding of functional cognate protein pairs (CifA and CifB). Afterward, to determine the individual gene activity, transgenic expression of a single *cif* gene can be conducted, allowing for the assessment of whether a gene alone can induce CI or whether other *cif* genes or factors are necessary for the CI to occur (Beckmann et al., 2017; Le Page et al., 2017; Adams et al., 2021; Horard et al., 2022).

The *w*Ltri Type III *cifA;B* are located adjacent to a conserved set of 11 genes, collectively known as the Undecim Cluster, which constitutes a eukaryotic association module (EAM) within phage WO. Phage WO, a bacteriophage that infects intracellular *Wolbachia*, faces the challenge of 2-fold barriers: the eukaryotic cell membrane and the intracellular bacterial cell membranes. Consequently, it frequently carries an EAM containing genes that exhibit eukaryotic-like functions and origins, which have the potential to influence host-*Wolbachia* interactions (Bordenstein and Bordenstein, 2022). After infection, the phage WO that integrates its genetic material into the *Wolbachia* genome refer as putative prophage WO. Although it is thought that no complete prophage WO has been identified in *w*Ltri, the genes surrounding the *cif*s exhibit module synteny akin to that of the WO-like island found in *w*No, *w*Mau, and *w*AlbB. These WO-like Islands are considered defective prophages, likely stemming from an ancestral prophage WO genome, which has since undergone domestication by the bacterial host or is undergoing degradation and elimination from the chromosome (Bordenstein and Bordenstein, 2022).

The WO-like island of *w*Ltri also exhibits module synteny with that of *w*Meg, a *Wolbachia* strain found in the blowfly *Chrysomya megacephala*, which is commonly associated with carrion and other decaying materials in human environments (Badenhorst and Villet, 2018). This similarity extends to the intergenic region between their Type III *cifA;B* and *terL*. However, the *terL* was distinct from the known *terL* genes of phage WO. Within *Wolbachia* genomes, *terL* is not exclusively associated with prophage WO but is also linked to Gene Transfer Agents (GTAs), which are virus-like structures responsible for packaging and transferring prokaryotic DNA between donor and recipient prokaryotic cells (Lang and Beatty, 2000; Lang et al., 2017; Bordenstein and Bordenstein, 2022). Although the *terL* in *w*Meg exhibited low nucleotide sequence similarity to a terminase-like gene in RcGTA of *R. capsulatus*, it demonstrated higher similarities to a terminase found in a putative GTA from *w*Mel (AE017196). This observation suggests that the *terL* in *w*Meg might be a component of GTAs within *Wolbachia* genomes. Furthermore, the *terL* homologs formed a distinct clade, separating them from other *terL* genes within the prophage WO region, suggests that the *terL* genes in *w*Meg and prophage WO have different evolutionary origins. This clade further branched into sub-clades that distinguished Supergroups A and B, consistent with previous finding that *terL* genes within putative GTAs in *Wolbachia* genomes can effectively differentiate between these Supergroups (Bordenstein and Bordenstein, 2022).

Regarding the potential synteny of the intergenic region between the *cif* genes and *terL* in the *Wolbachia* genomes, it appears to involve at least two “genomic scars” resulting from ancestral transposition events associated with IS256, IS110, IS982, IS5, and IS630. In *w*Meg, an ancient phage WO carrying Type III *cifA;B* might had integrated its genome into or near a GTA sequence. Subsequently, the GTA and the prophage WO may have deteriorated over time, or transposition events could have joined the breakpoints near the *cif* genes and *terL*, ultimately leading to the genetic remnants that are presently observed. Given that the intergenic sequences were frequently located in the vicinity of breakpoints in *w*Ltri and other *Wolbachia* genomes, the observed similarities in this region are likely a consequence of the latter phenomenon.

In summary, our bacterial community survey indicates that *Wolbachia* is the main endosymbiont in *L. trifolii*, alongside minor occurrences of *Acinetobacter*, *Pseudomonas*, and *Limnobacter*. The *Wolbachia* strain in *L. trifolii*, *w*Ltri, possesses three distinct types of cytoplasmic incompatibility factor (*cif*) genes: Type I, Type III, and Type V *cifA;B*. The diversification and cumulative presence of these genes may contribute to the strong CI effects observed in *L. trifolii*.

## Data availability statement

The data presented in the study are deposited in the DNA Data Bank of Japan (DDBJ) repository, accession number DRA017213 (https://ddbj.nig.ac.jp/resource/bioproject/PRJDB16160).

## Ethics statement

The manuscript presents research on animals that do not require ethical approval for their study.

## Author contributions

AP: Conceptualization, Data curation, Investigation, Validation, Writing – original draft, Writing – review & editing. AH: Conceptualization, Investigation, Writing – original draft, Writing – review & editing. YT: Funding acquisition, Project administration, Supervision, Writing – original draft, Writing – review & editing. HA: Funding acquisition, Project administration, Supervision, Writing – original draft, Writing – review & editing.
