# Supplementary figures and images for: Bacterial community and genome analysis of cytoplasmic incompatibility-inducing Wolbachia in American serpentine leafminer, Liriomyza trifolii
Source: Front Microbiol. 2024 Feb 6;15:1304401. doi: 10.3389/fmicb.2024.1304401 (PMC10877061; doi:10.3389/fmicb.2024.1304401)

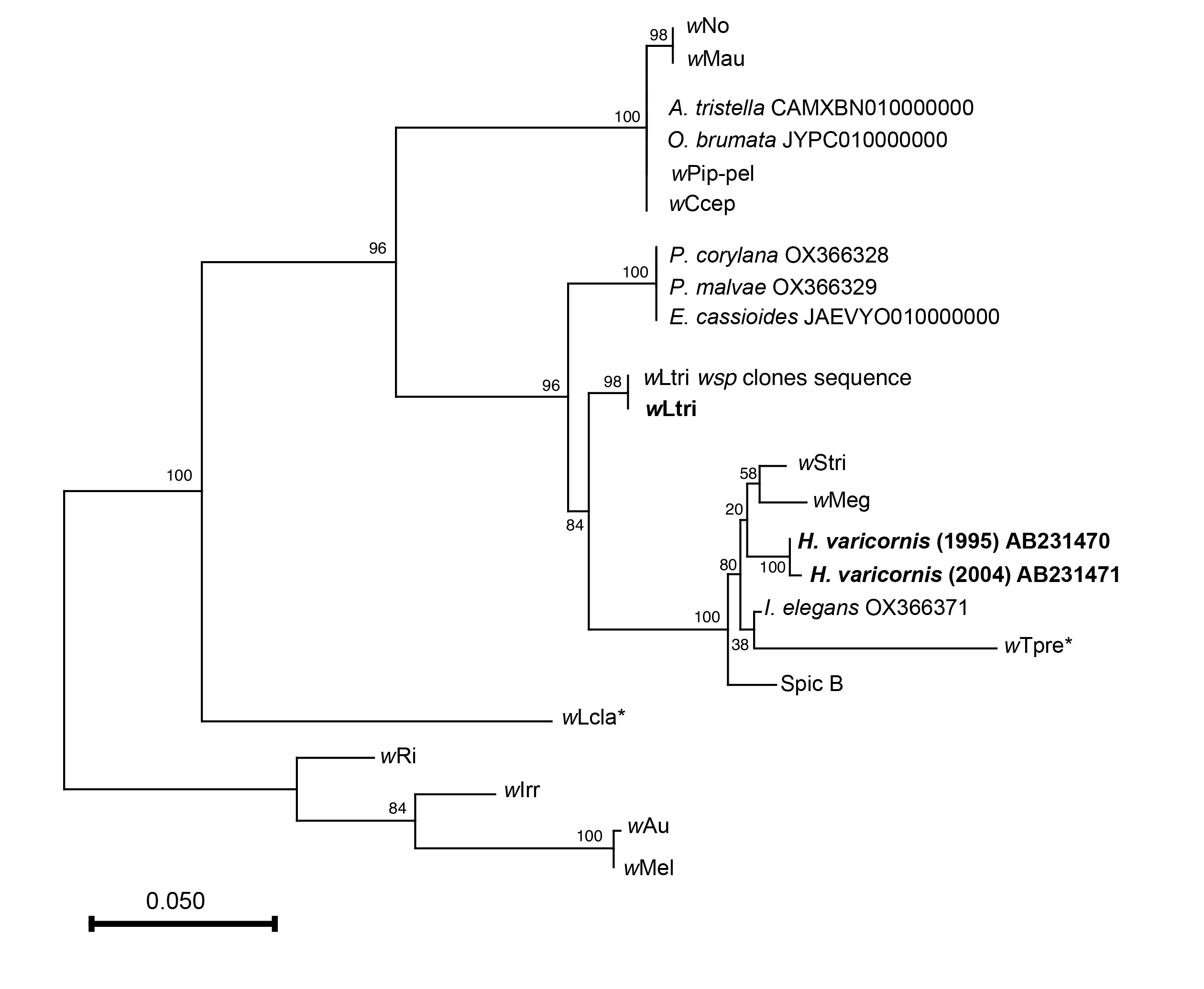

Supplement: Supplementary file 5 [file Image_1.JPEG]

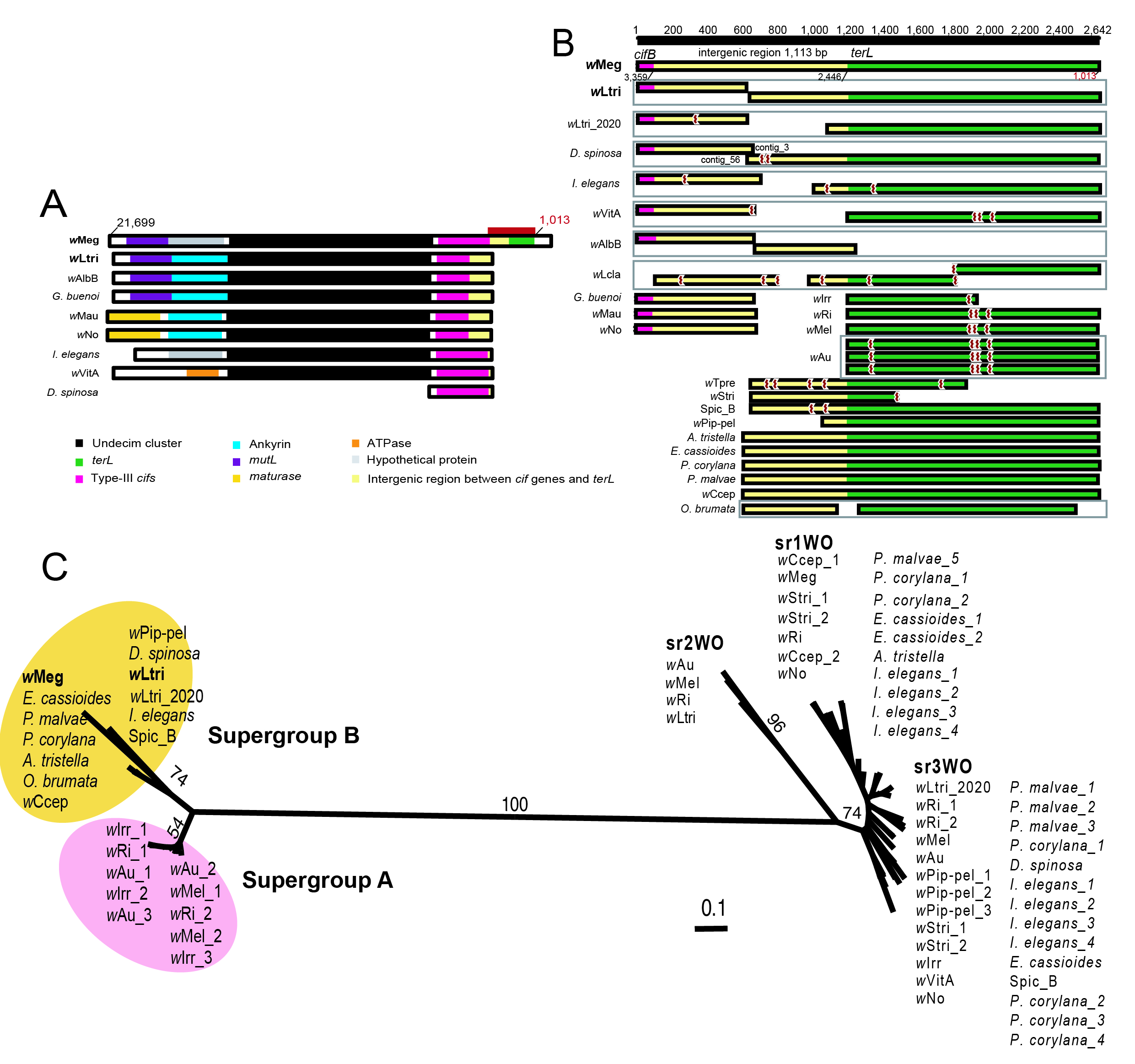

Supplement: Supplementary file 6 [file Image_2.JPEG]

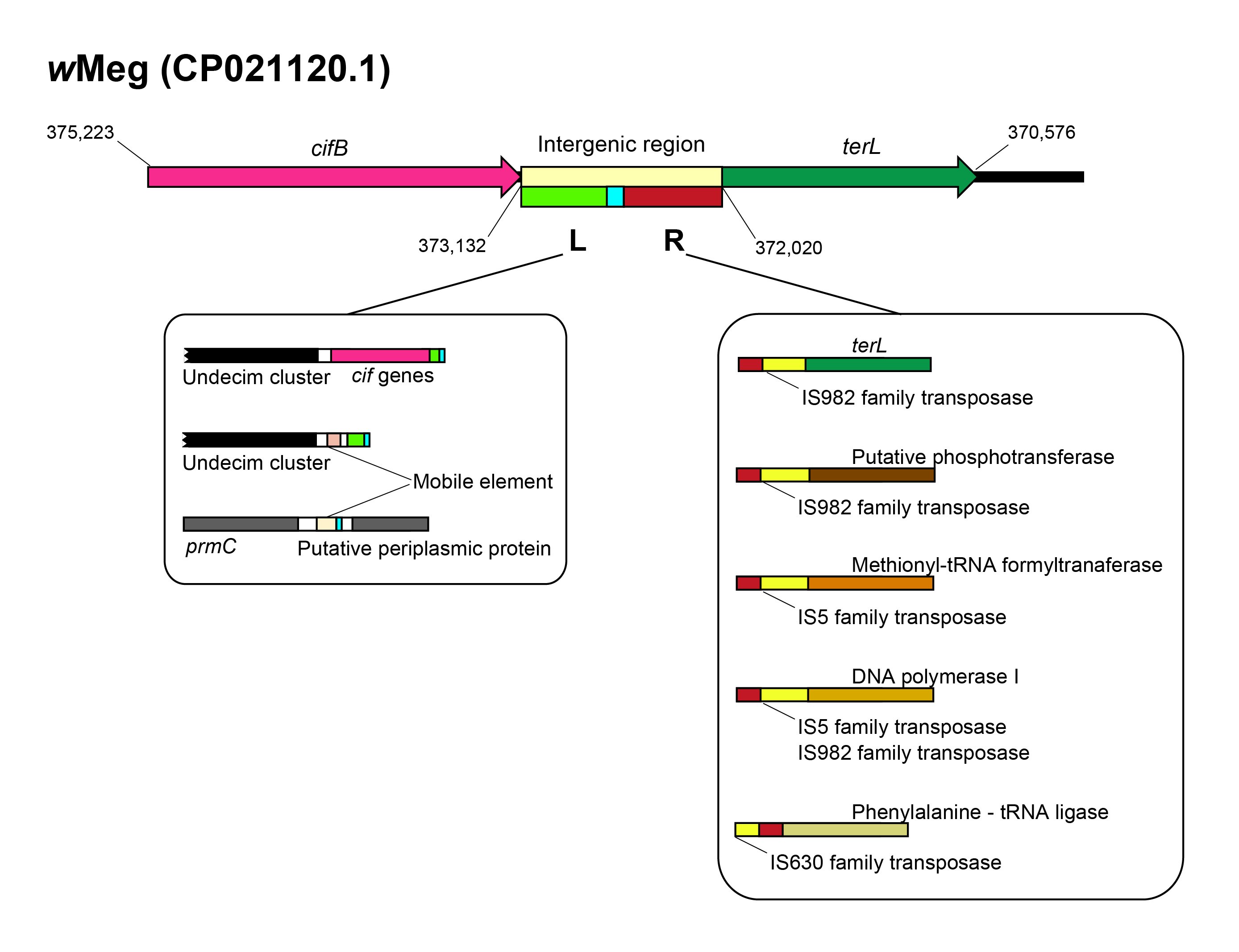

Supplement: Supplementary file 7 [file Image_3.JPEG]
